# Diffuse Alveolar Hemorrhage Caused by Disseminated Cryptococcosis in a Patient With Systemic Lupus Erythematosus

**DOI:** 10.7759/cureus.53831

**Published:** 2024-02-08

**Authors:** Takao Nagashima, Hiroki Yabe, Toshiaki Ogishi, Tsuyoshi Kobashigawa

**Affiliations:** 1 Division of Rheumatology, First Department of Comprehensive Medicine, Saitama Medical Center, Jichi Medical University, Saitama, JPN

**Keywords:** immunocompromised hosts, beta-d glucan, systemic lupus erythematosus, fungal infection, cryptococcosis, alveolar hemorrhage

## Abstract

A teenage girl with systemic lupus erythematosus (SLE) was admitted with fever, dry cough, and dyspnea on exertion. Chest computed tomography revealed bilateral diffuse infiltration and swelling of the mediastinal lymph nodes. The bronchoalveolar lavage (BAL) fluid was light red, suggesting diffuse alveolar hemorrhage (DAH). Therefore, glucocorticoid pulse therapy was initiated. However, blood and BAL fluid cultures showed the growth of *Cryptococcus neoformans*. The patient was diagnosed with disseminated cryptococcosis. The patient was treated with liposomal amphotericin B* and *flucytosine; the prednisolone dose was rapidly tapered. Infections should be thoroughly ruled out in patients with SLE and DAH.

## Introduction

Diffuse alveolar hemorrhage (DAH) is an uncommon but serious complication of systemic lupus erythematosus (SLE). The incidence of DAH in patients with SLE ranges from approximately 0.6% to 5.4%, and the mortality rate is highly variable, ranging from 0% to 92%, according to previous studies [1]. Hemoptysis can occur in less than half of the patients [2]. Infections should be ruled out as a differential diagnosis as they can be associated with DAH; however, it is often challenging to determine whether infections occurred at the time of DAH diagnosis [2].

Cryptococcosis is an infection caused by the yeast-like fungus Cryptococcus species. It often develops into an opportunistic infection in immunocompromised individuals. The respiratory and central nervous systems are also commonly affected. Patients with SLE often present with meningitis or disseminated cryptococcosis. The prognosis of cryptococcal meningitis or disseminated cryptococcal infection is poor in patients with SLE, resulting in death in up to one-third of the affected patients [3-5]. Pulmonary manifestations are often nonspecific and include fever, cough, sputum, dyspnea, and chest pain [6].

Hemoptysis is a symptom of cryptococcosis observed in up to 12% of patients [6,7]. However, DAH due to cryptococcal infection is rare [8]. Herein, we report the case of a patient with SLE complicated by DAH in the absence of hemoptysis, which is a manifestation of disseminated cryptococcosis.

## Case presentation

A teenage girl was admitted to our hospital with fever, dry cough, and dyspnea upon exertion. The patient had been diagnosed with SLE and biopsy-proven lupus nephritis IV-S (A) three months earlier and had been treated with prednisolone 50 mg/day plus mycophenolate mofetil. Before referral, levofloxacin was administered by a physician but with no effect.

**Figure 1:**
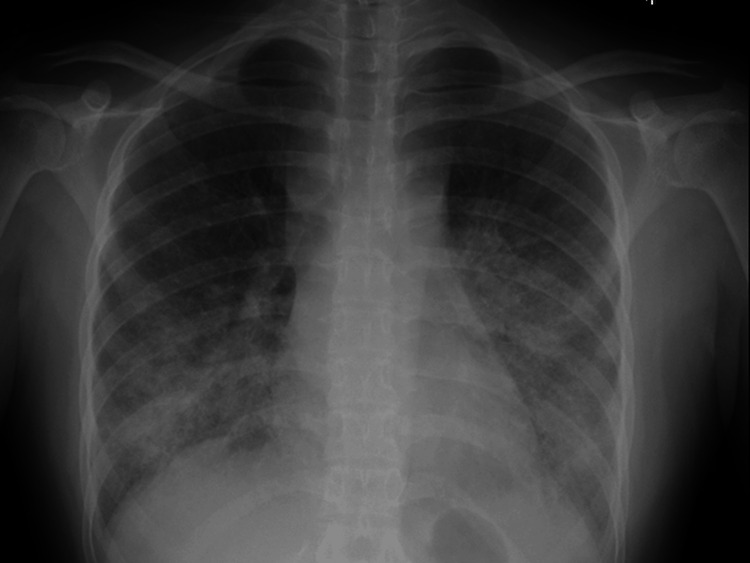
Chest radiograph Chest radiograph showing bilateral diffuse opacities with lower lung distributions.

**Figure 2:**
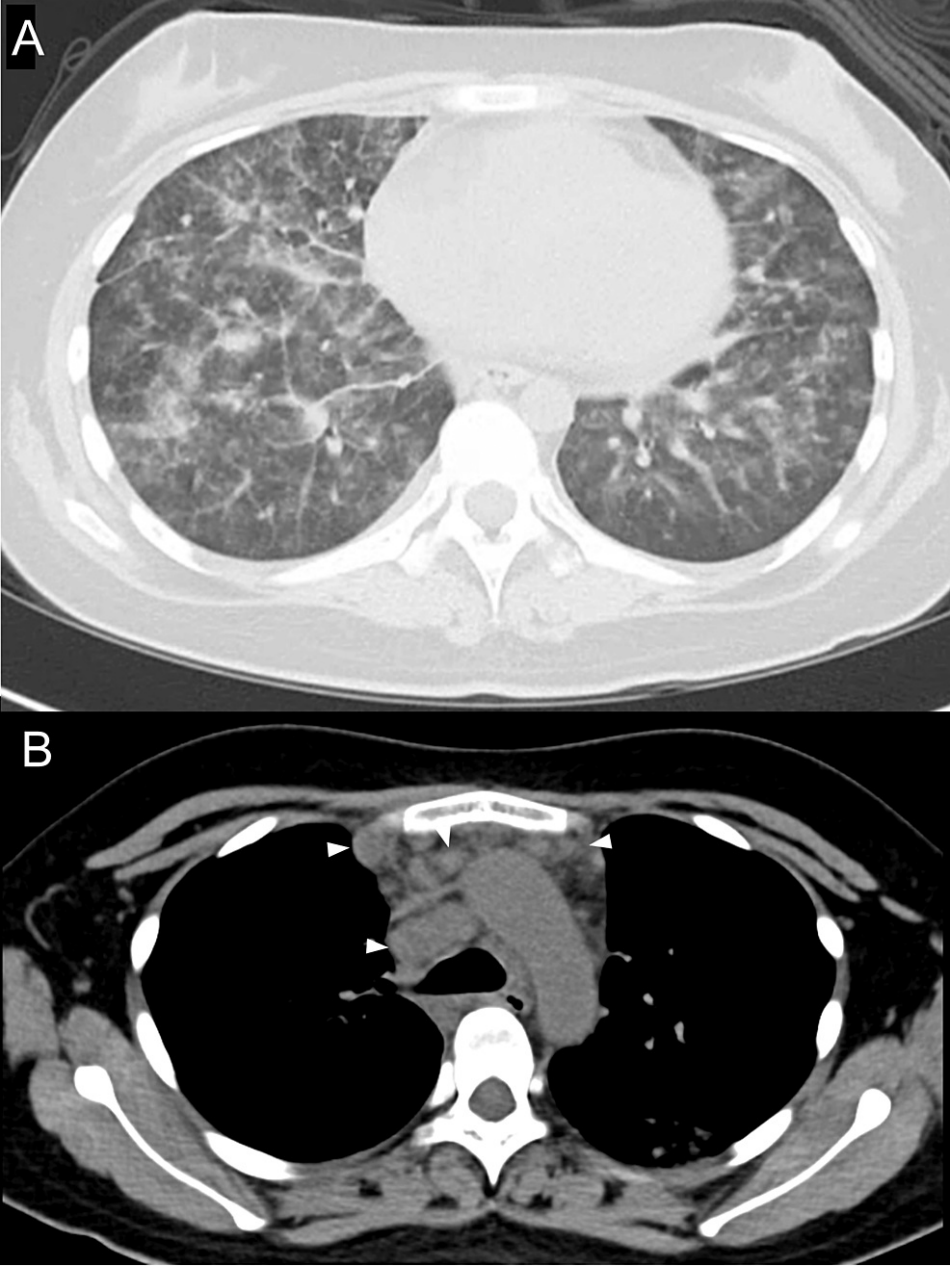
Computed tomography of the chest A. Computed tomography of the chest revealed diffuse infiltrations with focal consolidation. There were no nodular lesions or cavitation. B. The swelling of multiple mediastinal lymph nodes was conspicuous on mediastinal window views (arrows).

On admission, the patient was administered oral prednisolone (22.5 mg/day) and mycophenolate mofetil (1500 mg/day). On examination, the body temperature was elevated (40°C), while there was no crackle on chest auscultations. The laboratory findings were as follows (Table 1): leukocyte count was 7,800/µL (lymphocyte, 1%), and C-reactive protein was 15.5 mg/dL. The serum beta-D glucan level was within the normal range. Chest radiography revealed bilateral ground-glass infiltrates mainly in the lower field (Figure 1). Chest computed tomography (CT) revealed bilateral reticular infiltration of the lung field and swelling of the mediastinal lymph nodes (Figures 2A, 2B). Chest CT obtained three months ago revealed no swelling of the mediastinal lymph nodes.

**Table 1:** Laboratory findings on admission

Blood analyses	Result	Reference range
Hemoglobin (g/dL)	8.5	11.3-15.2
Leukocyte (×10^9^/L)	7.8	3.5-9.1
Neutrophil (%)	98	40-74
Lymphocyte (%)	1	19-48
Platelet count (×10^9^/L)	282	130-369
Blood urea nitrogen (mg/dL)	14	8-20
Creatinine (mg/dL)	0.65	0.46-0.79
Lactate dehydrogenase (U/L)	336	124-222
C-reactive protein (mg/dL)	15.5	<0.14
IgG (mg/dL)	843	870-1,700
C3 (mg/dL)	109	65-135
C4 (mg/dL)	25	13-35
Anti-dsDNA antibody (IU/mL)	10	<10
Beta-D glucan (pg/mL)	19.2	<20

Treatment with azithromycin was initiated based on a provisional diagnosis of atypical pneumonia. On the second day, bronchoscopic examination was performed. The three sequential bronchoalveolar lavage (BAL) fluids revealed a persistent light red color, suggesting DAH. Hemosiderin-containing macrophages were detected in the BAL fluid specimens. Although the disease activity of lupus nephritis seemed to be stable, the alveolar hemorrhage was thought to be caused by SLE. The patient was treated with intravenous pulsed methylprednisolone (1 g/day) for three days, followed by oral prednisolone (50 mg/day) and intravenous tazobactam/piperacillin. However, the fever decreased only transiently with pulse therapy, and the fever again reached over 40°C while on prednisolone. No improvement was observed on chest radiography. Subsequently, we found that the two sets of blood cultures obtained on admission grew Cryptococcus neoformans. Furthermore, the culture of the BAL fluid grew the same fungus, and the India ink test for the cerebrospinal fluid was positive. The patient was diagnosed with disseminated cryptococcosis. Treatment with liposomal amphotericin B in combination with flucytosine was initiated, and the prednisolone dose was quickly reduced to 20 mg/day. The respiratory condition, fever, and chest radiographic findings improved gradually. The patient was discharged after undergoing intravenous liposomal amphotericin B and flucytosine therapy for four weeks. The patient was advised a long-term fluconazole therapy.

## Discussion

Disseminated cryptococcosis caused DAH in a patient with SLE. Blood, BAL fluid, and cerebrospinal fluid cultures revealed Cryptococcus neoformans. In addition to bilateral diffuse infiltration in the lung field, swelling of the mediastinal lymph nodes was conspicuous on the chest CT.

In this case, DAH was induced by invasive cryptococcosis. Bronchoscopy-detected DAH is rare in cryptococcosis [8]. The causes of DAH can be characterized as infectious and noninfectious causes [9]. An association between infections and DAH has also been reported in immunocompromised patients. Pathogens include viruses; fungi, such as *Aspergillus spp., Candida spp., and Pneumocystis jirovecii*; bacteria, including *Mycoplasma spp.*; and mycobacteria [9]. However, cryptococcosis is not usually considered a cause of DAH.

Swelling of the mediastinal lymph nodes was noted on chest CT. Lymphadenopathy is not a common radiographic finding in DAH and is more suggestive of infection and malignancy [10]. The radiological features of cryptococcosis are nonspecific and diverse. Swelling of the hilar or mediastinal lymph nodes has been reported as a radiographic finding of cryptococcosis [11]. Several reports have described swelling of mediastinal and/or hilar lymph nodes as the presenting features of invasive or pulmonary cryptococcosis [12,13]. Swelling of the mediastinal lymph nodes is more commonly seen in immunocompromised patients than in immunocompetent patients, suggesting that the lymphatic spread of microorganisms may be more common in immunocompromised patients [11]. In cryptococcosis, enhanced CT may be useful for demonstrating moderate-to-marked enhancement of lymph nodes [14].

Although alveolar capillaritis has been suggested as the etiology of DAH in SLE, infection is another factor in the development of DAH [15,16]. One study demonstrated that BAL fluid culture was positive in more than half of patients with SLE complicated by DAH [17]. One case of infection by *Cryptococcus species *was included in this study, but it is challenging to differentiate between infection on admission and nosocomial infection [15]. In the case of DAH and fungal infections, it is often a terminal event due to nosocomial infection [15,18]. Although sputum, BAL fluid, and blood cultures are necessary to diagnose infections, these results are unavailable when treatment decisions must be made in clinical practice [19].

## Conclusions

We report a case of DAH caused by disseminated cryptococcosis in a patient with SLE. When DAH is encountered in an immunocompromised patient, infections should be carefully excluded. Multiple mediastinal or hilar lymph node swelling and diffuse pulmonary infiltrates may indicate disseminated cryptococcosis.
